# Supplementary material for: Peroxyacetic Acid Pretreatment: A Potentially Promising Strategy towards Lignocellulose Biorefinery
Source: Molecules. 2022 Sep 26;27(19):6359. doi: 10.3390/molecules27196359 (PMC9573572; doi:10.3390/molecules27196359)
Supplement: Supplementary file 1 [file molecules-27-06359-s001.zip › molecules-1921567-supplementary.pdf]

Supplementary Information

Peroxyacetic Acid Pretreatment: A Potentially Promising Strategy Towards Lignocellulose Biorefinery

Mingyang Hu, Junyou Chen†, Yanyan Yu, Yun Liu*†

(College of Life Science and Technology, Beijing University of Chemical Technology, Beijing 100029, China)

----

† These authors contributed equally to this work.

*Corresponding author (Yun Liu): Email: liuyun@mail.buct.edu.cn; or liuyunprivate@sina.com; Fax: +86-10-64416428; Tel: +86-10-64421335.

Figure captions

Figure S1. PAA 3D molecular structure (a) and chemical bonds and intramolecular hydrogen bond structures (b).

Figure S2. The mass balance of PAA pretreatment and saccharification process according the data from reference [49].

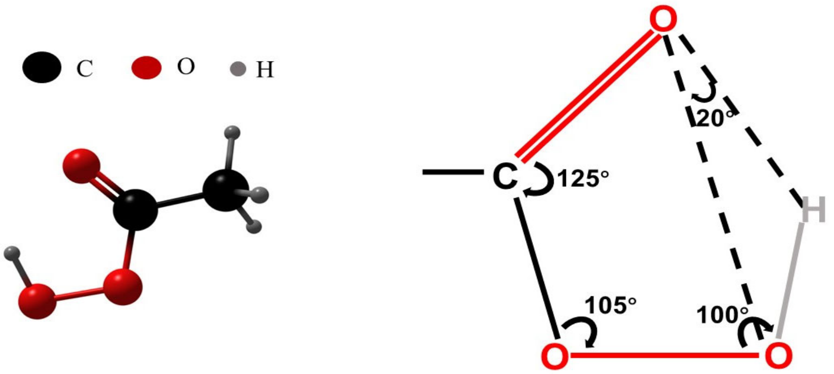

(a) (b)

Figure S1. PAA 3D molecular structure (**a**) and chemical bonds and intramolecular hydrogen bond structures (**b**).

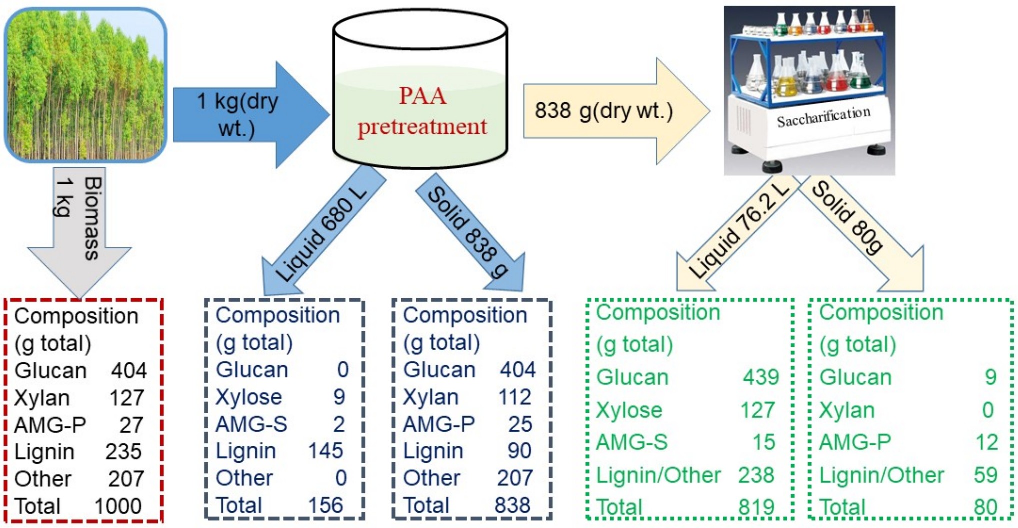

Figure S2. The mass balance of PAA pretreatment and saccharification process according to the data from reference [71]. One cycle of treatment with 125 mM NaOH followed by two cycles of PAA generated by perhydrolase and the subsequent saccharification. AMG-S: total content of arabinose, mannose and galactose. AMG-P: total content of arabinan, mannan, and galactan.
